# Si-Miao-Yong-An Decoction for Diabetic Retinopathy: A Combined Network Pharmacological and *In Vivo* Approach

**DOI:** 10.3389/fphar.2021.763163

**Published:** 2021-11-26

**Authors:** Ao Du, Yumin Xie, Hao Ouyang, Bin Lu, Wangya Jia, Hong Xu, Lili Ji

**Affiliations:** The MOE Key Laboratory for Standardization of Chinese Medicines, Shanghai Key Laboratory of Compound Chinese Medicines and The SATCM Key Laboratory for New Resources and Quality Evaluation of Chinese Medicines, Institute of Chinese Materia Medica, Shanghai University of Traditional Chinese Medicine, Shanghai, China

**Keywords:** Si-Miao-Yong-An decoction, Diabetic retinopathy, network pharmacology, inflammation, angiogenesis

## Abstract

Si-Miao-Yong-An decoction (SMYAD), a traditional Chinese medicine formula, is mainly used to clear away heat and detoxify and to promote blood circulation and relieve pain. Diabetic retinopathy (DR) is the most common type of microvascular complication caused by diabetes. This study is designed to examine the protective effect of SMYAD against DR and further to reveal the engaged mechanism *via* integrating network pharmacology and *in vivo* experimental evidence. Streptozotocin (STZ) was intraperitoneally injected into mice to induce diabetes. The dysfunction of the blood retina barrier (BRB) was observed by conducting Evan’s blue leakage assay, detecting tight junction (TJ) protein expression and counting the number of acellular capillaries in retinas. Our results showed that SMYAD alleviated BRB breakdown *in vivo*. Network pharmacology results demonstrated that regulating inflammation, immune responses, and angiogenesis might be associated with the efficacy of SMYAD in alleviating DR, in which the tumor necrosis factor (TNF) and hypoxia inducible factor 1 (HIF1) signal pathways were involved. Next, immunofluorescence staining results showed that SMYAD decreased microglia activation in retinas and reduced the enhanced adhesion of leukocytes into retinal vessels. SMYAD reduced the elevated serum TNFα content and retinal TNFα expression. SMYAD abrogated the activation of nuclear factor *κ*B (NF*κ*B) and HIF1*α* and consequently decreased the enhanced expression of some pro-inflammatory molecules and vascular endothelial growth factor (VEGF) in retinas. These results indicate that SMYAD attenuated DR development through suppressing retinal inflammation and angiogenesis *via* abrogating NFκB-TNFα and HIF1*α*-VEGF signal pathways.

## Introduction

Diabetic retinopathy (DR), a common microvascular complication caused by diabetes, has become the most serious eye disease leading to the adult blindness in the world (Antonetti et al., 2012; Gardlik and Fusekova, 2015). From 2015 to 2019, the prevalence rate of diabetic macular edema (DME) and DR was 27.0% in the world, among which the early period non-proliferative DR (NPDR) accounted for 25.2%, the later period proliferative DR (PDR) was 1.4%, and DME was 4.6% (Thomas et al., 2019). In China, the pooled prevalence rates in people with diabetes between 1990 and 2007 were 18.45, 15.06, and 0.99% for DR, NPDR, and PDR, respectively (Song et al., 2018). Therefore, more attention shall be paid to DR prevention.

At present, DR therapy is mainly based on drug treatment, laser photocoagulation treatment, and pars plana vitrectomy. Laser photocoagulation is an invasive treatment, which can cause postoperative visual field defects, decreased contrast sensitivity, and some other adverse reactions including central dark spots or para-central dark spots (Oellers and Mahmoud, 2016). These side effects greatly limited the clinical application of laser photocoagulation. The vascular endothelial growth factor (VEGF) antagonist, intravitreally injected when applied, is the most common drug used for DR treatment (Bandello et al., 2013). However, the application of the VEGF antagonist only is incapable of curing DR completely. Moreover, it has some side effects such as causing fibrovascular constriction and the sustained elevation of intraocular pressure (Singh and Kim, 2012; Bandello et al., 2013). Therefore, more drugs with better efficacy and fewer side effects need to be found in the future.

According to the theory of traditional Chinese medicine, the pathogenesis of DR was mainly due to the poor blood circulation and stasis caused by Yin deficiency and fire vigour that burned blood vessels. Recently, many traditional Chinese medicines (TCMs) and their active ingredients have been reported to improve DR. These TCMs include *Lonicera japonica* Thunb., *Salvia miltiorrhiza* Bge., *Lycium barbarum* L., *Andrographis paniculata* (Burm.f.) Nees, *Panax notoginseng* (Burkill) F.H.Chen, *Dendrobium officinale* Kimura and Migo, etc., and they all have functions like eliminating heat, improving blood circulation, nourishing Yin, and dissolving blood stasis (Behl and Kotwani, 2017; Vasant More et al., 2017; Xu et al., 2018).

Si-Miao-Yong-An decoction (SMYAD), first recorded in “*YanFangXinBian”* (Qing Dynasty), is composed of *Scrophularia ningpoensis* Hemsl. (Chinese name Xuan-Shen), *Lonicera japonica* Thunb (Chinese name Jin-Yin-Hua), *Angelica sinensis* (Oliv.), Diels (Chinese name Dang-Gui), and *Glycyrrhiza uralensis* Fisch. ex DC. (Chinese name Gan-Cao). It is traditionally applied externally for treating sore, carbuncle, and ulcer. Recently, SMYAD is generally used for the treatment of peripheral vascular diseases such as thromboangiitis obliterans. Among these four TCMs included in SMYAD, Jin-Yin-hua is good at clearing away heat and detoxifying, Xuan-Shen nourishes Yin and relieves fire, Dang-Gui is good at activating blood and dissipating blood stasis, and Gan-Cao reconciles various drugs. Therefore, SMYAD can be used to clear away heat and detoxify, to improve blood circulation, and to relieve pain, which implies its huge potential in DR treatment. Actually, a previous clinical study has demonstrated that SMYAD improved DR and some other diabetic vascular complications in diabetic patients (Zhang et al., 2012). However, the mechanism participating in the SMYAD-provided protection against DR is still not clear.

The pathogenesis of DR is very complex, which implies that modulating multiple targets is required for its treatment. Network pharmacology is generally used to clarify the relationship between the drug’s action and molecular targets (Hopkins, 2008). The therapeutic action of the Chinese medicinal formula may be obtained from the joint action of all compounds in the formula, so its pharmacological activity has multi-effects, multi-pathways, and multi-targets, which is consistent with the core theory and method of network pharmacology (Wang et al., 2021). Recently, network pharmacology is commonly used for the prediction of bioactive ingredients and the clarification of molecular targets in TCM’s research work (Li et al., 2014; Jiao et al., 2021; Wang et al., 2021).

The alleviation of SMYAD on DR was evaluated *in vivo*, and then, the potential engaged mechanism was investigated by integrating network pharmacology prediction and further *in vivo* experimental validation in this study.

## Materials and Methods

### Reagents

*Scrophularia ningpoensis* Hemsl., *Lonicera japonica* Thunb., *Angelica sinensis* (Oliv.) Diels, and *Glycyrrhiza uralensis* Fisch. ex DC. were purchased from Kangqiao Co., Ltd. (Shanghai, China). These four TCMs were authenticated by Professor Hong Xu, and voucher samples were kept in the Institute of Chinese Materia Medica (Shanghai University of Traditional Chinese Medicine). Chlorogenic acid (Jin-Yin-Hua), luteoloside (Jin-Yin-Hua), harpagide (Xuan-Shen), harpagoside (Xuan-Shen), ferulic acid (Dang-Gui), glycyrrhizinic acid (Gan-Cao), and liquiritin (Gan-Cao) (purity≥98%) were bought from Dalian Meilun Biotechnology Co., Ltd. (Dalian, China). The intercellular adhesion molecule 1 (ICAM1) antibody (ABclonal, Shanghai, China), the ionized calcium-binding adapter molecule 1 (Iba1) antibody (GeneTax Inc., Alton Parkway Irvine, CA), nuclear factor κB (NFκB) p65, *ß*-actin, Lamin B1, phosphorylated IκB kinase (p-IKK), the phosphorylated NFκ-B inhibitor (p-IκB), VEGF receptor 2 (VEGFR2), and the phosphorylated VEGFR2 antibodies (Cell Signaling Technology, Danvers, MA) were obtained. Tumor necrosis factor-α (TNFα), claudin1, claudin19, hypoxia inducible factor 1α (HIF1*α*), and vascular cell adhesion protein 1 (VCAM1) antibodies (Santa Cruz, Santa Cruz, CA), Alexa Fluor 568 goat anti-rabbit IgG (BD Biosciences, Franklin Lakes, NJ), and peroxidase-conjugated goat anti-rabbit immunoglobulin G (IgG) (H + L) and anti-mouse IgG (H + L) (Jackson ImmunoResearch, West Grove, PA) were obtained. BCA Protein Assay Kits and NE-PER nuclear and cytoplasmic extraction reagents were bought from Thermo Fisher Scientific (Waltham, MA). Enzyme-linked immunosorbent assay (ELISA) kits were from R and D (Minneapolis, MN). PrimeScript RT Master Mix and SYBR Premix Ex Taq kits (Takara, Shiga, Japan) were obtained. Fluorescein isothiocyanate-coupled concanavalin A (FITC-ConA) was bought from Vector Laboratories (Burlingame, CA). 4’,6-Diamidino-2-phenylindole (DAPI) and Trizol were purchased from Life Technology (Carlsbad, CA). Other reagents were bought from Sigma Chemical Co. (St. Louis, MO).

### Preparation of SMYAD Powder

The SMYAD powder was prepared as recorded in classic literature reports. The mixed *Scrophularia ningpoensis* Hemsl., *Lonicera japonica* Thunb., *Angelica sinensis* (Oliv.) Diels, and *Glycyrrhiza uralensis* Fisch. ex DC. (The ratio of weight is 3:3:2:1) were heated under reflux for 2 h with 10 times the amount of distilled water. The residue was heated under reflux again under the same condition after the extracts were filtered, and this procedure was repeated three times. The filtered extracts were concentrated and further freeze-dried into fine powder with the final yield of 36.5%.

### High-Performance Liquid Chromatography Analysis

High-performance liquid chromatography (HPLC) was performed on a Shiseido C18 column (250 nm × 4.6 mm, 5 μm, 30°C). The wavelength was set at 260 nm. The mobile phase consisted of a mixture of acetonitrile–0.05%H_3_PO_4_–Et_3_N (at 0–5 min, 0.2:99.8, v/v; at 5–15 min, increased from 0.2:99.8 to 3:97, v/v; at 15–30 min, 7:93, v/v; at 30–45 min, 8:92, v/v; at 45–65 min, 6:94, v/v; at 65–67 min, 10:90, v/v; at 67–70 min, 11:89, v/v; at 75–90 min, 12:88, v/v; at 90–115 min, 17:83, v/v; at 115–130 min, 20:80, v/v; at 130–135 min, 23:77, v/v; at 135–145 min, 30:70, v/v; at 145–175 min, 50:50, v/v) with a flow rate of 1.0 ml/min. Seven reference compounds including chlorogenic acid, luteoloside, harpagide, harpagoside, ferulic acid, glycyrrhizinic acid, and liquiritin were dissolved in methanol. The peak of seven main compounds in SMYAD was identified by comparing the retention time with that of reference compounds. Additionally, the contents of those above seven compounds in SMYAD were detected by using the external standard method.

### Experimental Animals

Specific-pathogen-free male C57BL/6 mice (20 ± 2 g) were obtained from the Shanghai Laboratory Animal Center of Chinese Academy of Science (Shanghai, China) and fed under a controlled room temperature (22°C ± 1 °C) and humidity (65 ± 5%) with a 12:12 h light/dark cycle. Mice have free access to food and water and have received humane care according to the institutional animal care guidelines approved by the Experimental Animal Ethical Committee of Shanghai University of Traditional Chinese Medicine (approved number: PZSHUTCM200724021).

### Animal Treatment

A total of 45 mice were intraperitoneally injected (ip) with 55 mg/kg BW of streptozotocin (STZ) (Sigma No. S0130) in citric acid solution and 0.1 M trisodium citrate solution pH = 4.4 (vehicle) for 5 consecutive days. Also, other 15 mice in the control group were injected with physiological saline. Seven days later, mice with a blood glucose concentration over 16.7 mmol/L were considered as diabetic mice and included in this study. These diabetic mice were randomly divided as follows: DM, DM + SMYAD (1.5 g/kg), and DM + SMYAD (4.5 g/kg). Each group has 15 mice. One month later, SMYAD (1.5, 4.5 g/kg, intragastric administration) was orally given to mice for 1 consecutive month, and then, mice were sacrificed to collect blood samples and eyes. The body weight was monitored and the blood glucose concentration was determined with a Accu-Check glucometer (Roche Diagnostics, Germany) every 7 days during the whole experimental process.

### Evan’s Blue Leakage Assay

Blood-retina-barrier (BRB) breakdown was detected according to the published method (Mei et al., 2019; Zhang et al., 2019). Briefly, mice were injected with 2% Evan’s blue (10 μl/g, i. p.). Two hours later, retinas were dissected after the Evan’s blue dye was completely removed from retinal vessels. Formamide was used to dissolve the retinas, and the absorbance in the extracts was determined at 620 nm. The amount of Evan’s blue dye was calculated, and the results were normalized to the dried weight of the retina.

### Leukostasis Assay

Leukostasis was detected as previously described (Chen et al., 2019). Briefly, FITC-ConA (120 μl) was intravenously injected into mice. Twenty minutes later, the isolated eyes were fixed in a 4% paraformaldehyde (PFA) solution. Retinas were dissected and flat-mounted for the observation under an inverted microscope (IX81, Olympus, Japan). FITC-ConA + cells adhered to retinal blood vessels were counted.

### Hematoxylin-Eosin Staining of Acellular Capillaries in Retinas

The retina was fixed in a 4% PFA solution for 24 h and gently rocked overnight after four times of cleaning with ultrapure water and then digested with 3% trypsin and stained with hematoxylin-eosin (H and E) the next day. The morphology of retinal vessels was pictured by using an inverted microscope.

### Network Pharmacology Analysis

Network pharmacology analysis was conducted as described in the guidance (Li et al., 2021).

Target Collection: Active targets of SMYAD were acquired from BATMAN-TCM (http://bionet.ncpsb.org.cn/batman-tcm/) and TCMSP (http://tcmspnw.com/) databases. According to the suggested criterion, compounds were selected if the drug-likeness was ≥0.18 and the oral bioavailability was ≥30%. Additionally, the indicator chemical compounds recorded in Chinese Pharmacopoeia from four Chinese botanical drugs included in SMYAD (Chinese Pharmacopoeia Commission, 2020) with low drug-likeness and oral bioavailability values were also retained in this study.

Protein–Protein Interaction (PPI) Network Construction: Diabetic Retinopathy (ICD-11, 9B71.0Z) therapeutic targets were collected by using GeneCards (www.genecards.org/). Cytoscape 3.6.1 software was used to construct a drugs–ingredients–targets network. A target-to-target and function-related PPI network was established *via* the STRING database.

KEGG and GO Analysis: DAVID 6.8 was used to analyze the KEGG pathway enrichment and GO function of the proteins involved in the PPI network. The R package of ggplot2 was used for enrichment analysis, and the cutoff value was set as *p* < 0.05.

### Immunofluorescence Staining

Retinal Iba1 immunofluorescence staining was conducted according to the published method (Mei et al., 2019; Zhang et al., 2019), but in this study, retinas were incubated with the Alexa fluor 568 goat anti-rabbit IgG (H + L) antibody after incubating with the Iba1 antibody overnight.

### qPCR Analysis

Retinal RNA was extracted, cDNA was synthesized, and real-time RT-PCR was conducted as described in kits. The relative expression of target genes was normalized to actin, analyzed by the 2^−ΔΔCt^ method, and calculated as the ratio compared with the control. The primer sequences are shown in Table 1.

**TABLE 1:** List of primers for real-time PCR.

Target	Primer	Sequence
TNFα	Forward	5′- ATG​TCT​CAG​CCT​CTT​CTC​ATT​C-3′
	Reverse	5′- GCT​TGT​CAC​TCG​AAT​TTT​GAG​A-3′
ICAM1	Forward	5′- CTG​AAA​GAT​GAG​CTC​GAG​AGT​G-3′
	Reverse	5′- AAA​CGA​ATA​CAC​GGT​GAT​GGT​A-3′
MCP-1	Forward	5′- CAA​GAG​TGA​ATC​CAC​ACA​ACA​G -3′
	Reverse	5′- GTA​GGA​GTC​AAC​TCA​GCT​TTC​T -3′
HIF1*α*	Forward	5′- GAA​TGA​AGT​GCA​CCC​TAA​CAA​G -3′
	Reverse	5′- GAG​GAA​TGG​GTT​CAC​AAA​TCA​G -3′
VEGF	Forward	5′- TAG​AGT​ACA​TCT​TCA​AGC​CGT​C -3′
	Reverse	5′- CTT​TCT​TTG​GTC​TGC​ATT​CAC​A -3′
Actin	Forward	5′- TTC​GTT​GCC​GGT​CCA​CAC​CC -3′
	Reverse	5′- GCT​TTG​CAC​ATG​CCG​GAG​CC -3′

### Western-Blot Analysis

Proteins were extracted, and the protein concentration was determined. The denatured samples were run on SDS-PAGE gels and electrophoretically transferred to PVDF membranes that were further incubated with primary and secondary antibodies. The protein expression was visualized by using a chemiluminescent reagent and quantified by calculating gray densities of the target protein blots with internal controls.

### ELISA

Serum contents of TNFα, monocyte chemoattractant protein-1 (MCP-1), interleukin-6 (IL-6), and VEGF were detected by using commercial ELISA kits.

### Statistical Analysis

Data are shown as the mean ± standard error of the mean (SEM). The statistical significance of differences between groups is obtained through one-way ANOVA with the LSD post hoc test. *p* < 0.05 is considered to be the significant difference.

## Results

### Chemical Characterization of SMYAD

**FIGURE 1:**
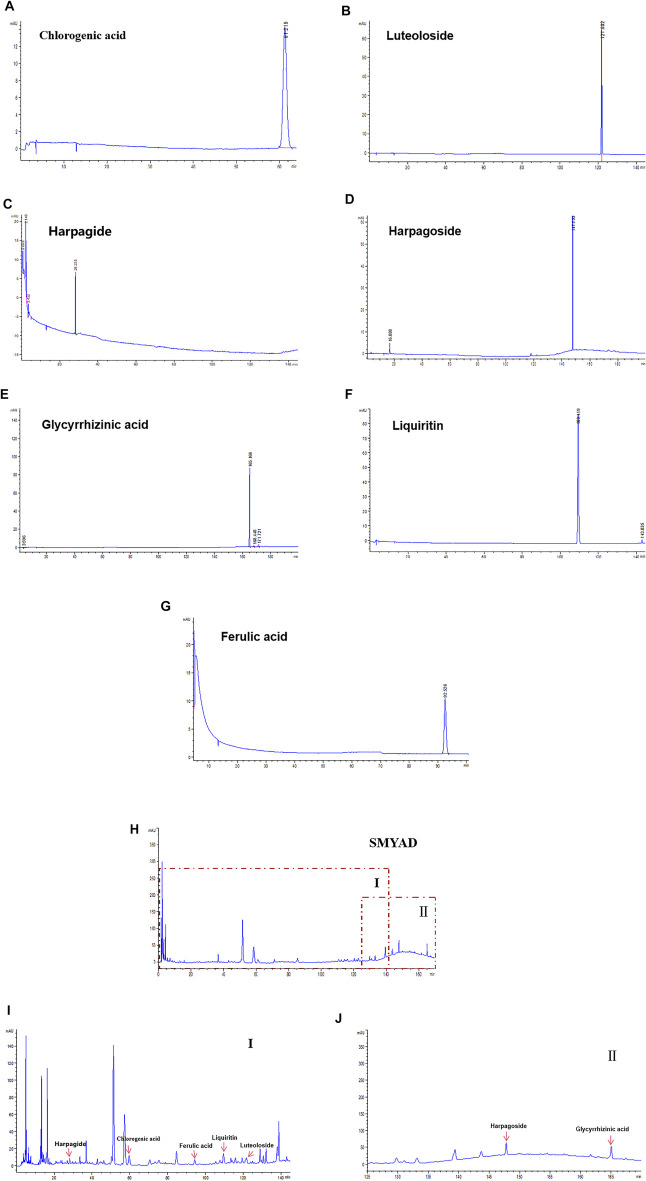
HPLC analysis. HPLC chromatogram of reference compounds including **(A)** chlorogenic acid, **(B)** luteoloside, **(C)** harpagide, **(D)** harpagoside, **(E)** glycyrrhizinic acid, **(F)** liquiritin, and **(G)** ferulic acid. HPLC chromatogram of **(H)** SMYAD and **(I)** SMYAD in areaⅠand **(J)** SMYAD in areaⅡ.

Data in Figures 1A–G demonstrated the HPLC chromatogram of chlorogenic acid, luteoloside, harpagide, harpagoside, ferulic acid, glycyrrhizinic acid, and liquiritin. Figures 1H–J demonstrate the HPLC chromatogram of SMYAD, and the above seven compounds were pointed out in SMYAD. Next, the content of these above seven compounds in SMYAD was determined. After calculations, the content of chlorogenic acid, luteoloside, harpagide, harpagoside, ferulic acid, glycyrrhizinic acid, and liquiritin in SMYAD was 0.882, 0.034, 0.284, 0.103, 0.034, 0.258, and 0.201%, respectively.

### SMYAD Alleviated BRB Dysfunction *in vivo*

**FIGURE 2:**
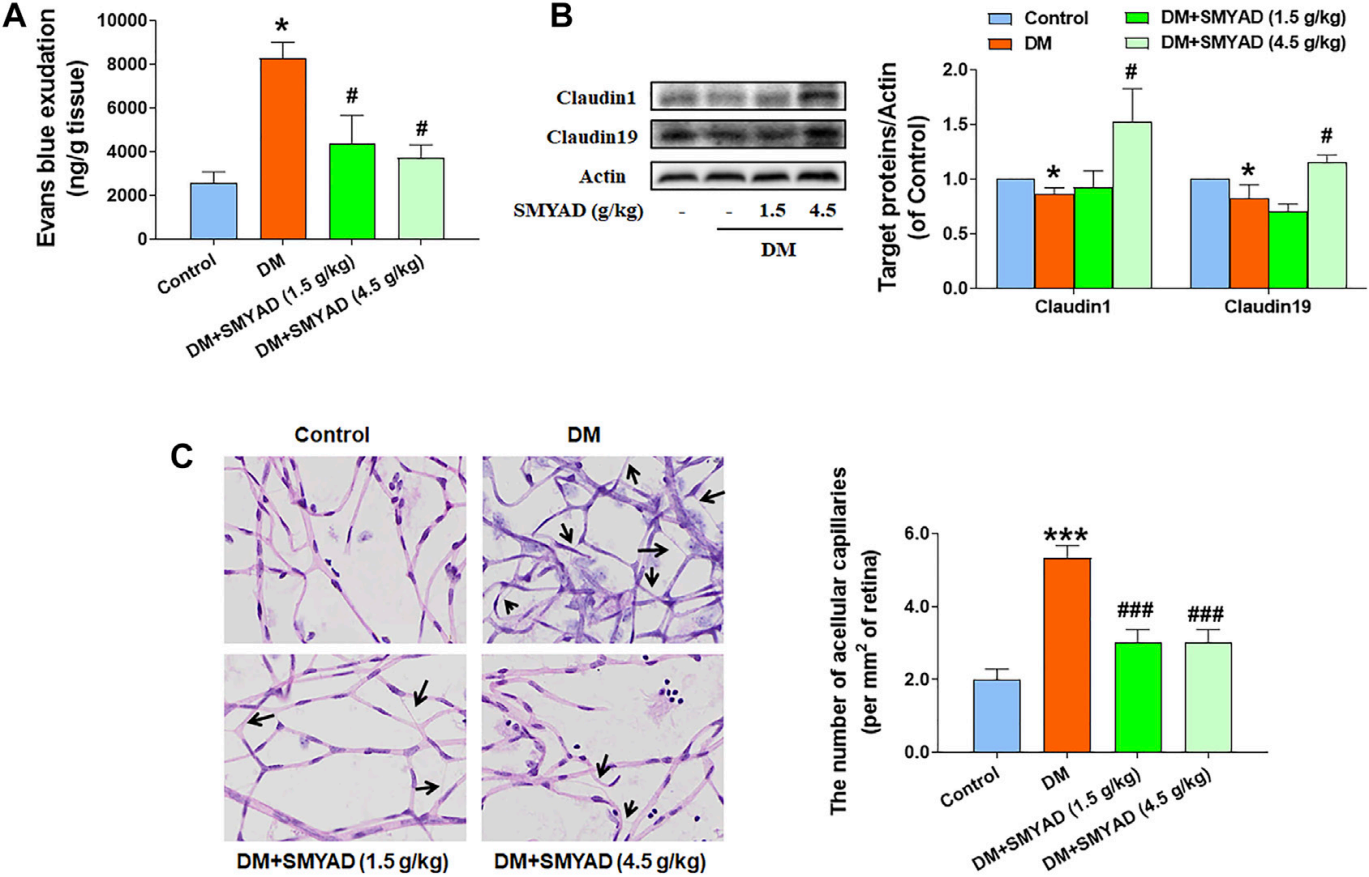
SMYAD alleviated BRB dysfunction *in vivo*. **(A)** Evan’s blue leakage assay (n = 4). **(B)** Claudin1 and claudin19 expression in retinas. The quantitative result is shown at right (n = 3). **(C)** Retinal H and E staining. The number of acellular capillaries per field in retinas is shown at right (n = 3). Black arrows indicate acellular capillaries. Data = mean ± SEM. *p < 0.05, ***p < 0.001 vs. control; ^#^ *p* < 0.05, ^###^ *p* < 0.001 vs. DM.

The increased blood glucose concentration and the reduced body weight of diabetic mice did not change after the administration of SMYAD (1.5, 4.5 g/kg) (data not shown). The elevated Evan’s blue dye leakage in retinas from diabetic mice was reduced after the administration of SMYAD (1.5, 4.5 g/kg) (Figure 2A). As shown in Figure 2B, the decreased expression of retinal claudin1 and claudin19 (two typical tight junctions, TJs) in diabetic mice was enhanced when diabetic mice received SMYAD (4.5 g/kg). Moreover, the decreased number of acellular capillaries marked with black arrows in retinas from diabetic mice was enhanced after the administration of SMYAD (1.5, 4.5 g/kg) (Figure 2C).

### Network Pharmacology Analysis

**FIGURE 3:**
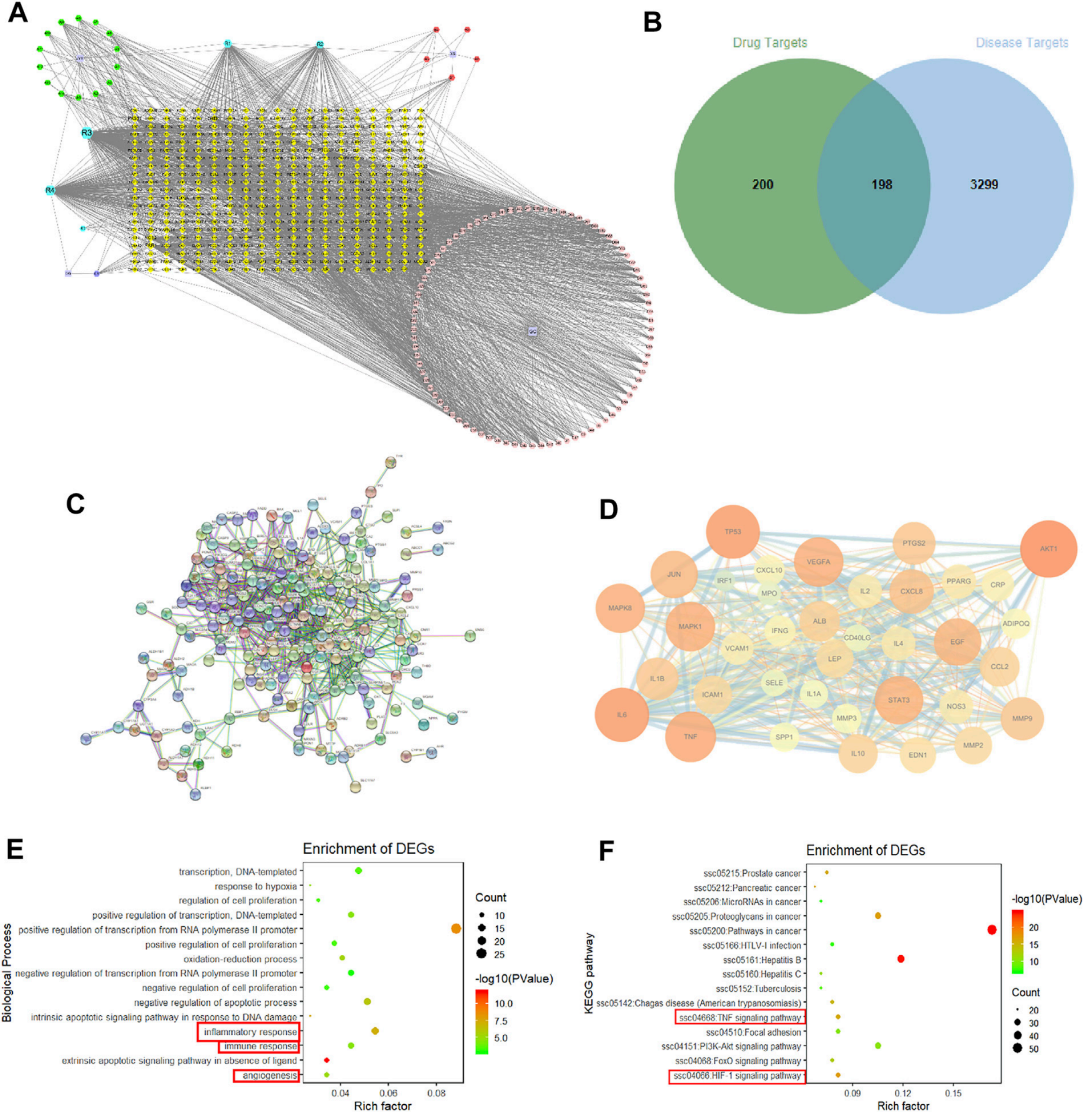
Network pharmacology. **(A)** Drugs–compounds–targets network. The node representing the compound is represented by a multi-color hexagon, and the target point is represented by a yellow diamond. **(B)** Venn diagram. **(C)** PPI network. **(D)** PPI network of core targets extracted from degree centrality. **(E)** GO enrichment analyses. A total of 15 biological process items in descending order of *p*-values in the DAVID database. **(F)** KEGG enrichment analyses.

A total of 109 active compounds and 398 compound-related targets were acquired from TCMSP and BATMAN-TCM. A DR target network was established to find the relationship between compounds in SMYAD and common targets of DR. As shown in Figure 3A, the yellow diamond-shape node in the central area represents the total 398 compounds-related targets, the green circular-shape node on the upper-left corner represents 14 active compounds from Jin-Yin-Hua, the red circular-shape node on the upper-right corner represents five compounds from Xuan-Shen, the purple circular-shape node on the bottom-left corner represents one compound from Dang-Gui, the pink circular-shape node on the bottom-right corner represents 84 compounds from Gan-Cao, and the blue circular-shape node represents five co-owned compounds from Jin-Yin-Hua, Xuan-Shen, Dang-Gui, and Gan-Cao. A total of 3,497 targets involved in DR development were obtained from the GeneCards database. After integrating the targets of compounds from SMYAD and the targets involved in DR development, 198 common matched-targets were found, and the Venn diagram is shown in Figure 3B. Next, those above 198 common matched-targets were imported into the STRING database to construct a PPI network. After removing 60 disconnected nodes, there were a total of 138 nodes (representing 138 common matched targets) and 817 edges (representing the interaction between two targets) (Figure 3C). After screening according to the degree of these nodes, the core targets are shown in Figure 3D. The node became larger and its color changed from yellow to red with the increased degree of the targets. Among these core targets, the first six molecular targets with higher degrees were protein kinase B (AKT1), IL-6, tumor protein p53 (TP53), TNF, mitogen-activated protein kinase 8 (MAPK8), and VEGFA.

These above 198 common matched-targets were further imported into the DAVID database, and a total of 960 GO items and 133 KEGG pathways were obtained. As shown in Figure 3E, inflammation (GO: 0006954), immune responses (GO: 0006955), and angiogenesis (GO: 0001525) marked with a red square accounted for a large proportion of biological processes. Moreover, the results of KEGG pathways showed that the TNF signaling pathway (hsa04668) (critically involved in inflammation and immune responses) and the HIF1 signaling pathway (hsa04066) (critically involved in angiogenesis) marked with a red square were the key signal pathways involved in the SMYAD-provided protection against DR.

Next, we consulted some transcriptome chips conducted in DR research included in GEO Databases (http://www.ncbi.nlm.nih.gov/gds/). The transcriptome chip results from mice at about 6 weeks after STZ injection (GSE111465) showed that the TNF signaling pathway marked with a red square was the most important signaling pathway involved in DR development in mice at 6 weeks after STZ treatment (Supplementary Figure S1A). Additionally, the transcriptome chip results from mice at about 12 weeks after STZ injection (GSE19122) showed that the VEGF signaling pathway marked with a red square was the most important signaling pathway involved in DR development in mice at 12 weeks after STZ treatment (Supplementary Figure S1B).

### SMYAD Alleviated Retinal Inflammation *in vivo*

**FIGURE 4:**
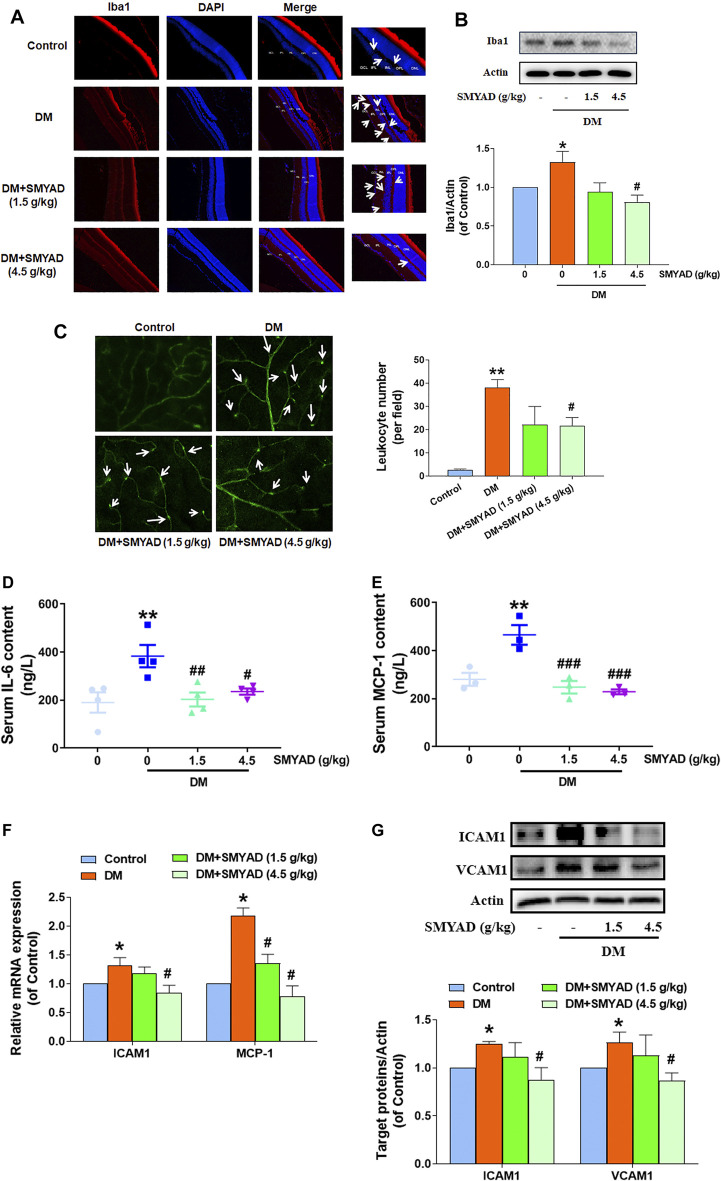
SMYAD alleviated retinal inflammation *in vivo*. **(A)** Representative images of retinal Iba1 immunofluorescence staining (scale bar: 20 mm). Enlarged images are shown at right (scale bar: 5 mm). White arrows indicate microglia. **(B)** SMYAD reduced the enhanced Iba1 expression in retinas. The quantitative result is shown below (n = 3). **(C)** Representative images of retinal FITC-ConA staining. The number of entrapped leukocytes in retinal vessels was calculated and is shown at right (n = 3). **(D)** Serum IL-6 amount (n = 4). **(E)** Serum MCP-1 amount (n = 3). **(F)** Retinal mRNA expression of ICAM1 and MCP-1 (n = 3). **(G)** SMYAD decreased the increased retinal expression of ICAM1 and VCAM1. The quantitative result is shown below (n = 3). Data = mean ± SEM. *p < 0.05, **p < 0.01 vs. control; ^#^ *p* < 0.05, ^##^ *p* < 0.01, ^###^ *p* < 0.001 vs. DM.

Studies have reported that Iba1 is a microglia/macrophage specific marker, which is widely used to label microglia (Kanazawa et al., 2002; Ibrahim et al., 2011). The enhanced number of Iba1-staining microglia (marked with white arrows) in retinas from diabetic mice was decreased after the administration of SMYAD (Figure 4A). The elevated retinal Iba1 expression in diabetic mice was decreased when diabetic mice received SMYAD (4.5 g/kg) (Figure 4B). The increased number of leukocytes adhered to the retinal capillary (marked with white arrows) from diabetic mice was also decreased after the administration of SMYAD (4.5 g/kg) (Figure 4C). Next results showed that the enhanced serum IL-6 and MCP-1 contents in diabetic mice were decreased when diabetic mice received SMYAD (1.5, 4.5 g/kg) (Figures 4D,E). The elevated ICAM1 mRNA expression in retinas from diabetic mice was decreased after the administration of SMYAD (4.5 g/kg), and the elevated retinal MCP-1 mRNA expression in diabetic mice was decreased after the administration of SMYAD (1.5, 4.5 g/kg) (Figure 4F). Moreover, the elevated protein expression of both ICAM1 and VCAM1 in retinas from diabetic mice was decreased when diabetic mice received SMYAD (4.5 g/kg) (Figure 4G).

### SMYAD Reduced TNFα Expression and Inhibited NFκB Activation *in vivo*

**FIGURE 5:**
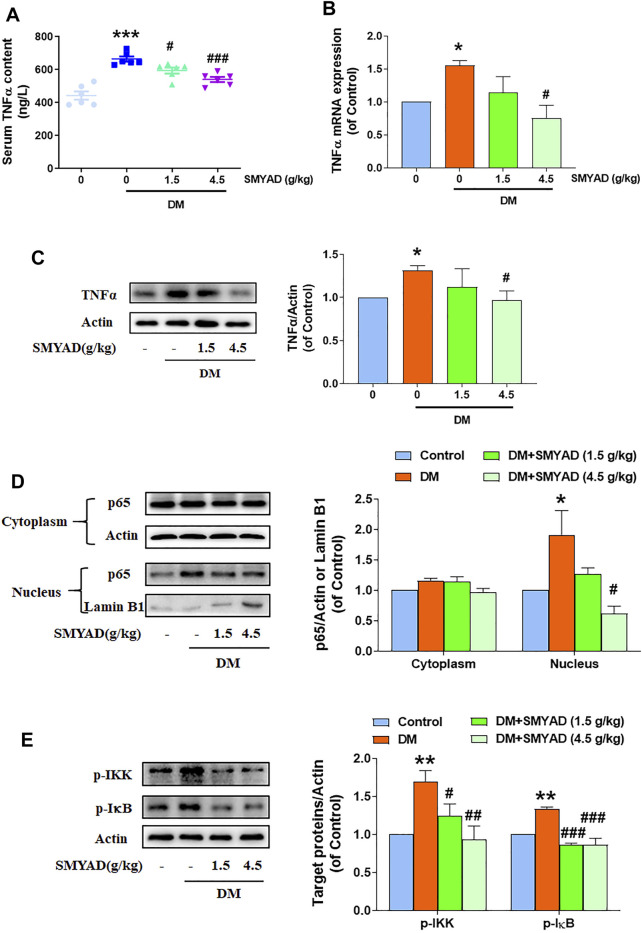
SMYAD reduced TNFα expression and blocked NFκB activation *in vivo*. **(A)** Serum TNFα content (n = 6). **(B)** Retinal mRNA expression of TNFα (n = 3). **(C)** SMYAD reduced the enhanced TNFα expression in retinas. The quantitative result is shown below (n = 3). **(D)** SMYAD abrogated the nuclear accumulation of NFκBp65 in retinas. The quantitative result is shown at right (n = 3). **(E)** SMYAD decreased the enhanced phosphorylation of IκB and IKK. The quantitative result is shown at right (n = 3). Data = mean ± SEM. *p < 0.05, **p < 0.01, ***p < 0.001 vs. control; ^#^ *p* < 0.05, ^##^ *p* < 0.01, ^###^ *p* < 0.001 vs. DM.

Data in Figure 5A showed that the enhanced serum TNF-α content in diabetic mice was obviously decreased when diabetic mice received SMYAD (1.5, 4.5 g/kg). Meanwhile, the enhanced retinal TNF-α mRNA and protein expression in diabetic mice was also decreased when diabetic mice received SMYAD (4.5 g/kg) (Figures 5B,C). Next results showed that SMYAD (4.5 g/kg) abrogated the nuclear accumulation of NFκ-B subunit p65 in retinas from diabetic mice (Figure 5D). Moreover, the elevated phosphorylation of IκB and IKK in retinas from diabetic mice was decreased when diabetic mice received SMYAD (1.5, 4.5 g/kg) (Figure 5E).

### SMYAD Inhibited the HIF1*α*-VEGF/VEGFR2 Signal Pathway *in vivo*

**FIGURE 6:**
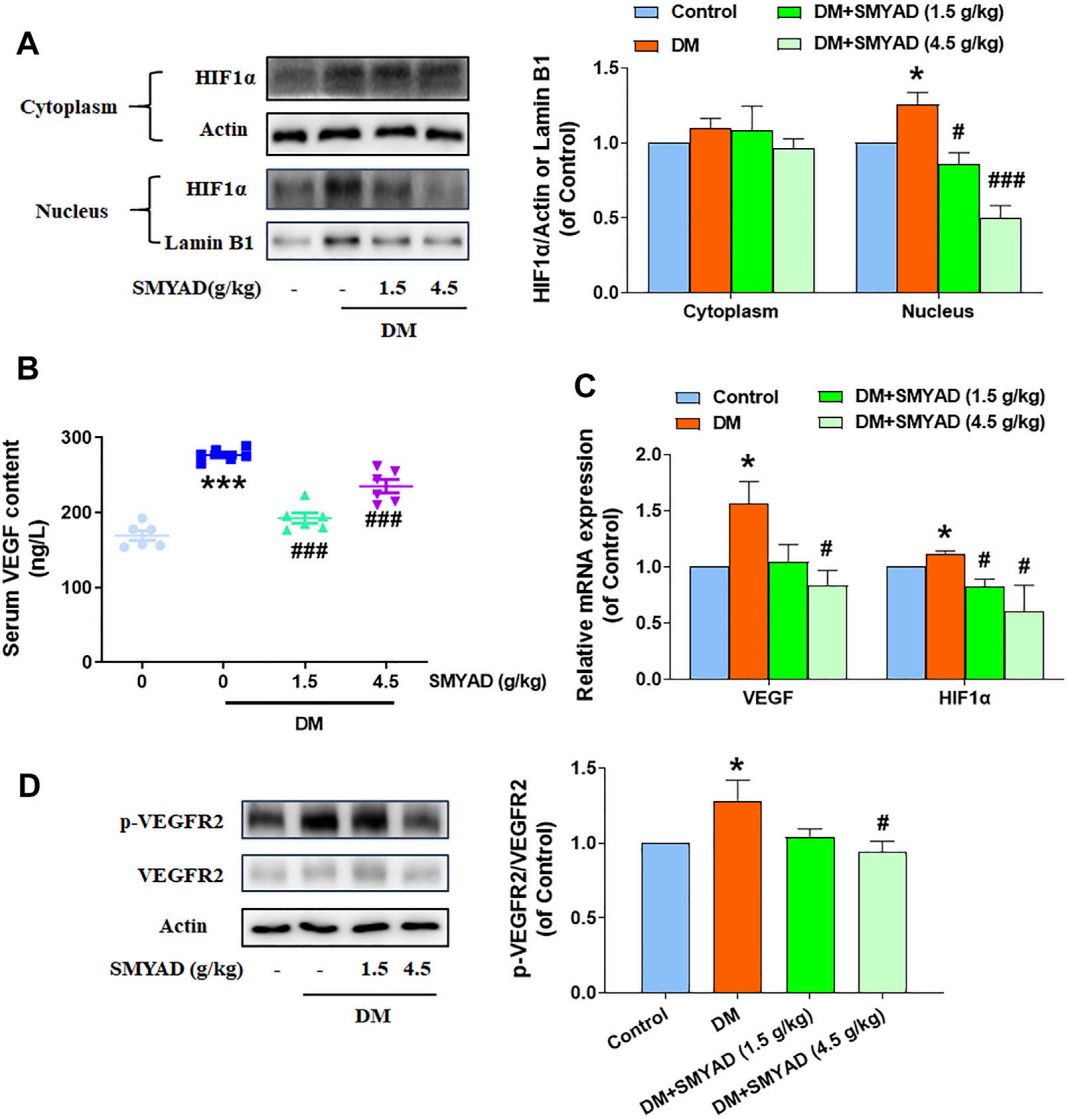
SMYAD inhibited the HIF1α-VEGF/VEGFR2 signal pathway *in vivo*. **(A)** SMYAD reduced nuclear accumulation of HIF1*α* in retinas. The quantitative result is shown at right (n = 3). **(B)** Serum VEGF content (n = 6). **(C)** HIF1*α* and VEGF mRNA expression in retinas (n = 3). **(D)** SMYAD decreased the elevated phosphorylation of VEGFR2. The quantitative result is shown at right (n = 3). Data = mean ± SEM. *p < 0.05, ***p < 0.001 vs. control; ^#^ *p* < 0.05, ^###^ *p* < 0.001 vs. DM.

SMYAD (1.5, 4.5 g/kg) abrogated the nuclear accumulation of HIF1*α* in retinas from diabetic mice (Figure 6A). Data in Figure 6B showed that the elevated serum VEGF content in diabetic mice was decreased after the administration of SMYAD (1.5, 4.5 g/kg). The increased retinal VEGF mRNA expression in diabetic mice was decreased when diabetic mice received SMYAD (4.5 g/kg) (Figure 6C). The enhanced retinal HIF1*α-*mRNA expression in diabetic mice was also obviously decreased when diabetic mice received SMYAD (1.5, 4.5 g/kg) (Figure 6C). Moreover, the enhanced VEGFR2 phosphorylation in retinas from diabetic mice was decreased when diabetic mice received SMYAD (4.5 g/kg) (Figure 6D).

## DISCUSSION

The dysfunction of BRB is commonly recognized as the earliest and typical event during DR progression (Zhang et al., 2014). BRB dysfunction is a crucial indicator for reflecting retinal microvascular injury in diabetic patients, which eventually causes severe visual impairment when no prompt intervention is applied (Rudraraju et al., 2020). Claudin1 and claudin19, two important TJs, greatly contributed to maintain the BRB integrity (Erickson et al., 2007). In this study, SMYAD reduced the leakage of Evan’s blue dye in retinas. SMYAD also restored the decreased retinal claudin1 and claudin19 expression *in vivo*. These above results indicate that SMYAD ameliorated BRB dysfunction *in vivo*. Acellular capillaries are a kind of microvessels with the blood vessel basal membrane and without endothelial cells. The occurrence of acellular capillaries was commonly used for reflecting the severity of retinopathy (Douglas et al., 2012; Chen et al., 2019). SMYAD decreased the enhanced number of acellular capillaries in retinas from diabetic mice. All these above results clearly demonstrated that SMYAD alleviated BRB dysfunction during DR progression.

Next, network pharmacology was performed to find the potential molecular targets involved in the SMYAD-provided alleviation on DR. Among those core molecular targets obtained from network pharmacology, the first six targets with higher degrees were AKT1, IL-6, TP53, TNF, MAPK8, and VEGFA, and they are all reported to regulate inflammation and angiogenesis (Kaštelan et al., 2020; Zhang et al., 2021; Zhou et al., 2021). Moreover, the enrichment results imply the crucial involvement of inflammation, immune responses, and angiogenesis in the SMYAD-provided protection against DR, as well as the potential participation of the TNF signaling pathway and HIF1 signaling pathway. Next, the results from two transcriptome chips conducted in DR research from GEO Databases further validated the previous experimental hints from network pharmacology that TNFα and HIF1*α*-VEGF signaling pathways participated in the progression of DR. The persistent low-grade inflammation caused by the elevated pro-inflammatory cytokines and other mediators was reported to be closely associated with the injury of retinal vasculature, which will cause BRB breakdown and induce retinal neovascularization during DR progression (Adamis, 2002; Aveleira, et al., 2010; Mesquida, et al., 2019). Therefore, retinal inflammation and neoangiogenesis are crucially involved in the whole course of DR. Our results from network pharmacology indicate that SMYAD may alleviate DR *via* inhibiting TNFα-mediated inflammation and HIF1*α*-initiated neoangiogenesis in retinas. Meanwhile, these prediction results from network pharmacology were further validated in the next following experiments.

Retinal inflammation initiated by the activated microglia is reported to be associated with DR progression (Baptista et al., 2017; Xu and Chen, 2017). This study showed that SMYAD reduced the enhanced Iba1 expression in retinas, suggesting that SMYAD inhibited retinal microglia activation *in vivo*. Leukostasis is a mild intra-vascular inflammatory response, and it is also an early event during DR progression reflecting retinal ischemia in diabetic patients (Adamis, 2002; Joussen et al., 2004). MCP-1 can recruit monocytes and macrophages, and its expression was elevated during DR development (Taghavi et al., 2019). ICAM1 and VCAM1 can recruit leukocytes to adhere to the endothelium and thus regulate leukostasis (Barouch et al., 2000). We found that SMYAD down-regulated MCP-1, ICAM1, VCAM1, and IL-6 expression and reduced leukostasis in retinas. These results suggest that SMYAD suppressed microglia activation and leukostasis in retina and thus alleviated retinal inflammation during DR development. Moreover, the results further validated the previous network pharmacology prediction that SMYAD alleviated BRB dysfunction during DR progression *via* inhibiting retinal inflammation.

As a main pro-inflammatory cytokine, TNFα was reported to regulate intra-ocular inflammatory responses and DR pathogenesis (Joussen, et al., 2002; Aveleira et al., 2010). TNFα was found to promote the adhesion of leukocytes to the endothelium in the retina and thus caused BRB damage (Joussen et al., 2002; Behl et al., 2008; Aveleira et al., 2010). As predicted in network pharmacology, the TNF signaling pathway participated in the SMYAD-provided amelioration on DR. Next experimental results further proved that SMYAD actually lowered the elevated retinal and serum TNFα contents. Transcription factor NFκB regulates the expression of many pro-inflammatory molecules including TNFα, IL-6, MCP-1, ICAM1, and VCAM1 (Shih et al., 2015). The NFκB signaling pathway is crucial for regulating retinal inflammation during DR progression (Kern, 2007). Our results found that SMYAD suppressed NFκB transcriptional activation and thus contributed to the reduced TNFα amount. All these will contribute to its inhibition on retinal inflammation, which shall be helpful for the alleviation of DR.

Previous network pharmacology results predicted the participation of angiogenesis and the important involvement of the HIF1 signaling pathway in the SMYAD-provided alleviation on DR. The dysfunction of the HIF1*α-*signaling pathway has already been found to be related with DR progression (Catrina and Zheng, 2021). Our experimental results further evidenced the inhibition of SMYAD on HIF1*α* activation in retinas *in vivo*. HIF1*α* can regulate the expression of VEGF, which drives neoangiogenesis *via* binding to its receptors, including VEGFR1, VEGFR2, and VEGFR3, and thus regulates pathological angiogenesis and the elevated vascular permeability during DR development (Witmer et al., 2003; Vriend and Reiter, 2016). SMYAD decreased the enhanced expression of VEGF and phosphorylated VEGFR2 in retinas. These above results suggest that SMYAD reduced retinal angiogenesis through suppressing the HIF1*α*-VEGF/VEGFR2 signaling cascade.

Our results demonstrated the alleviation of SMYAD on DR *in vivo* for the first time. After integrating the results from network pharmacology and the *in vivo* experimental validation, we found that SMYAD improved DR by inhibiting inflammation and angiogenesis through abrogating the NFκB and HIF1*α*-VEGF/VEGFR2 signal pathways. However, we must also recognize that the current network pharmacology database cannot yet contain all the signaling molecules involved in DR development, so there may be some other important molecules that have not been found in our study, which needs further deeper investigation in the future. This study provides an experimental basis for the clinical application of SMYAD in patients with DR in the future.

## Data Availability

The original contributions presented in the study are included in the article/Supplementary Material, and further inquiries can be directed to the corresponding authors.
