# Supplementary figures and images for: Si-Miao-Yong-An Decoction for Diabetic Retinopathy: A Combined Network Pharmacological and In Vivo Approach
Source: Front Pharmacol. 2021 Nov 26;12:763163. doi: 10.3389/fphar.2021.763163 (PMC8661904; doi:10.3389/fphar.2021.763163)

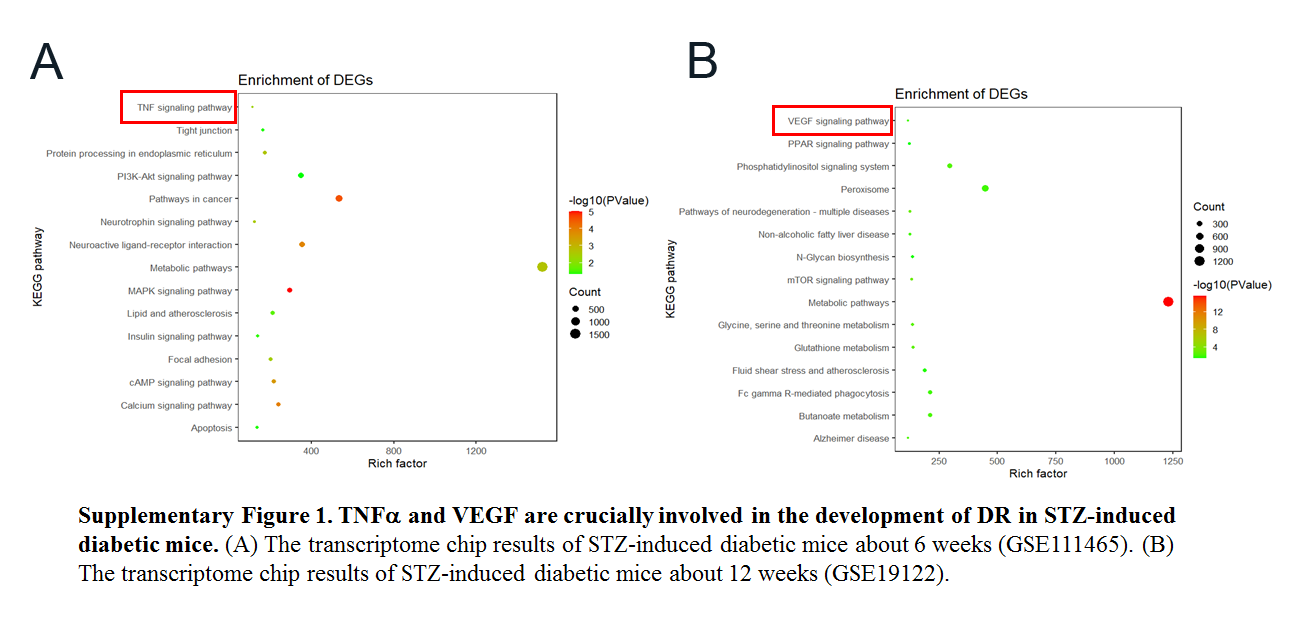

Supplement: Supplementary file 1 [file Image1.TIF]
